# Hemorrhagic Blisters, Necrosis, and Cutaneous Ulcer after Envenomation by the Niquim Toadfish

**DOI:** 10.4269/ajtmh.19-0321

**Published:** 2019-09

**Authors:** Vidal Haddad, Mônica Lopes-Ferreira, Adriana Lúcia Mendes

**Affiliations:** 1 Department of Dermatology, Faculty of Medicine, Sao Paulo State University, Botucatu, Brazil;; 2 Immunoregulation Unit of the Special Laboratory of Applied Toxinology, Butantan Institute, São Paulo, Brazil;; 3 Department of Internal Medicine, São Paulo State University, São Paulo, Brazil

**Figure 1.**
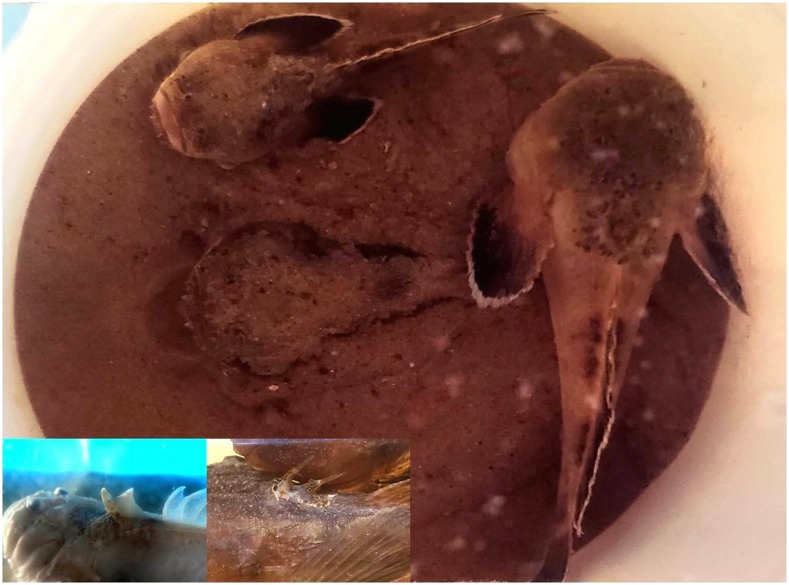
Live specimens of the toadfish *Thalassophryne nattereri*, with one of them semi-buried in the sand in a typical position. In the details, dorsal spicules of the fish, responsible for inoculation of the venom. Photos: Vidal Haddad Jr. This figure appears in color at www.ajtmh.org.

Toadfishes are found in tropical, marine, and estuarine waters. They have a highly developed venomous apparatus with dorsal and preopercular spines (Figure 1). Envenomation by this species can cause local inflammatory manifestations such as pain, edema, and erythema that can progress to cutaneous necrosis.^1–5^

**Figure 2.**
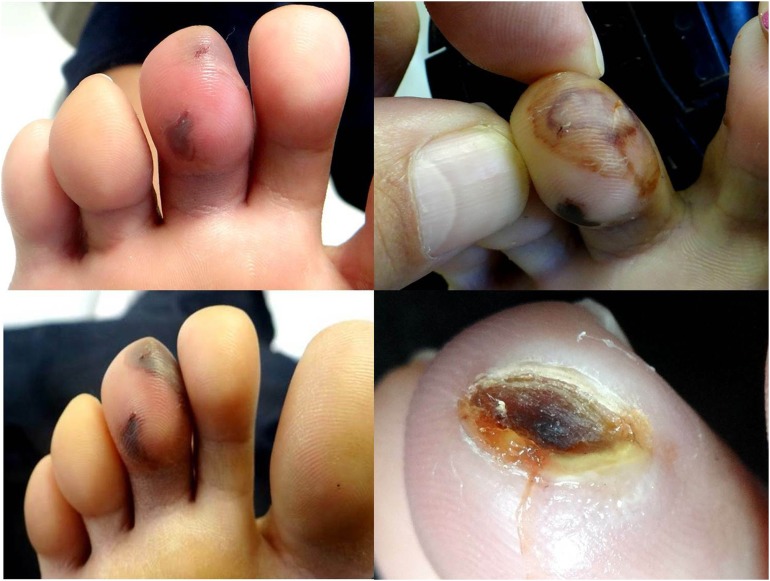
Evolution of patient envenomation, from the initial inflammation and hemorrhagic blistering (left) to the skin necrosis with ulcer (right). The process lasted about a month. Photos: Vidal Haddad Jr. This figure appears in color at www.ajtmh.org.

A 38-year-old woman stepped on something in a lagoon among the stones of a beach in Bahia state, Brazil. She then noticed two small perforations in the third toe of the right foot with slight bleeding. The place began to ache unbearably and she was medicated with painkillers. After 3 days, intense inflammation and hemorrhagic blisters appeared near to the perforations. In about 10 days, the upper blister delimited a necrosis and the formation of an ulcer covered by hemato-meliceric crust (Figure 2). The pain, which had persisted for about a week, had disappeared. One month later, ulcer was healed, leaving a scar.

Wounds by venomous fishes can be difficult to identify. Catfishes and stingrays cause mainly unique perforations, but the envenomation by toadfishes causes a characteristic double perforation by the dorsal spicules. The species present in the region is *Thalassophryne nattereri*, the “niquim.”^3–5^

The initial treatment is the immersion of the place in hot water for 30–90 minutes. Treatment with warm water minimizes pain intensity.^5^ Bacterial infection is common.^5^
